# Post-fledging habitat use in a declining songbird

**DOI:** 10.7717/peerj.7358

**Published:** 2019-08-30

**Authors:** Clayton D. Delancey, Kamal Islam

**Affiliations:** Department of Biology, Ball State University, Muncie, IN, United States of America

**Keywords:** Cerulean Warbler, Fledgling habitat use, *Setophaga cerulea*, Wood-warbler

## Abstract

**Background:**

Fledglings of many mature forest-dependent Neotropical songbirds move from mature forest habitats into areas of thick vegetation such as regenerating clearcuts. The Cerulean Warbler (*Setophaga cerulea*), a Neotropical migratory songbird, is a species of conservation concern across its range and it is listed as endangered in Indiana. This species has declined faster than any other species of wood-warbler in North America. Most prior research on Cerulean Warblers has examined the breeding biology, but there are no data on habitat use by fledgling Cerulean Warblers. Our research aimed to determine where fledgling Cerulean Warblers dispersed after they left their nest, but before they migrated to their wintering grounds.

**Methods:**

Since 2007, Cerulean Warbler breeding populations have been monitored in Yellowwood and Morgan–Monroe state forests in southern Indiana as part of a 100-year study called the Hardwood Ecosystem Experiment. To identify habitats used by fledgling Cerulean Warblers, we captured by hand or mist-nets, adult and juvenile Cerulean Warblers once young had fledged from a nest. We attached radio-transmitters to individuals and tracked each bird daily using radio-telemetry. Radio-telemetry data were collected from May to July 2015–2017, and microhabitat data on fledgling locations and random locations were collected during the same years in the month of July.

**Results:**

Fledgling presence, when compared to random non-use sites, was positively correlated to presence of grapevines, greater vertical vegetation density, and greater ground and canopy cover. Fledgling presence was negatively correlated with white oak abundance, aspect, basal area, and the abundance of mature trees that Cerulean Warbler adults use for nesting.

**Conclusions:**

Our study is the first to demonstrate that Cerulean Warbler fledglings occupy habitats that are characterized by specific habitat components. Fledgling sites were located in areas with high vegetation density, such as clusters of grapevine, which provided cover from predators. Identifying Cerulean Warbler habitats throughout the breeding season can better inform natural resource personnel on how to manage forests to meet the habitat needs of this rapidly declining migratory songbird.

## Introduction

Over the last several decades, many species of birds have declined as forested habitats have been altered or destroyed by humans. As a consequence, many species are listed as threatened. Therefore, it is imperative that we closely manage and preserve the remaining habitats for these species (Martin & Finch, 1995; Robinson et al., 1995; Holmes, 2007; Sauer et al., 2012). Many songbird studies focus on breeding habitat with a focus on adult birds, but leave out a large part of a songbird’s annual cycle, the fledgling period (Robinson et al., 1995; Campbell, Witham & Hunter Jr, 2007; Bakermans, Rodewald & Vitz, 2012). To best protect and manage habitats for a particular species, it is vital to understand a species’ entire life cycle. Based on a handful of studies, many species shift in their use of habitats between the breeding and post-breeding/fledgling periods (e.g., Streby et al., 2011; Porneluzi et al., 2014; Burke, Thompson III & Faaborg, 2017).

The Cerulean Warbler (*Setophaga cerulea*) is considered one of the fastest declining Neotropical wood-warblers in North America (Sauer et al., 2012). It is a small, migratory songbird that breeds in forests of the central and eastern United States (USFWS, 2006). According to the Breeding Bird Survey (BBS), its population has declined more than 75% from 1966 to 2006 and it is now considered a species of conservation concern by the U.S. Fish and Wildlife Service (Buehler et al., 2008). Cerulean Warblers are also listed as species of international concern and are listed as endangered in Canada (COSEWIC, 2010). Birdlife International in partnership with the International Union for Conservation of Nature (IUCN) classifies Cerulean Warblers as ‘vulnerable’ (BirdLife International, 2019). In Indiana, Cerulean Warblers are endangered (Indiana General Assembly, 2007). Species trend information, based on BBS data collected from 1966 to 2012, indicate that Cerulean Warblers are declining at about 3% per year. Declines in Cerulean Warbler populations have been attributed to habitat losses on their breeding and wintering grounds (Weakland & Wood, 2005; Buehler et al., 2008).

Research on the breeding ecology of Cerulean Warblers has been conducted in some parts of its distribution. However, this species has been a challenge for researchers because it nests and forages high up in the canopy. To date, very little research has been conducted on juvenile survivorship, fledgling movements, or pre-migratory activity. Until recently, it was difficult to follow individuals (Hamel, Dawson & Keyser, 2004). However, with new technology such as radio-transmitters and geolocators that are small enough for use on small Passerines, researchers can now explore this aspect of its ecology. According to Hamel (2000a, 2000b), unknown aspects of Cerulean Warbler biology are the behaviors and habitat preferences exhibited by this species during the fledgling period on its breeding grounds before migration.

The objective for this study was to better understand fledgling movements. Specifically, we were interested in identifying habitats used by fledglings. We chose to analyze microhabitat variables from locations where fledgling Cerulean Warblers were found and compared these data to microhabitat variables from random non-use locations. For the first time, we discuss fledgling Cerulean Warbler habitat characteristics, and offer forest management recommendations.

## Materials & Methods

### Study area

The Hardwood Ecosystem Experiment (HEE) is a large-scale, 100-year study that examines the effects of forest management on plant and animal species. This project is established in southern Indiana in the Morgan–Monroe and Yellowwood state forests within nine study units. There are three control units, three units with even-aged forest management, and three units with uneven-aged forest management (Kalb & Mycroft, 2013). Research cores are areas where treatments were applied; outside of the research cores lies a 50 m buffer where no harvest will occur. At the control sites, no harvest or forest management took place for the duration of the study. Clearcuts and shelterwood cuts characterize the even-aged units, and the uneven-aged units receive both single-tree and group cut harvests. The research core of the even-aged units consists of 4 ha openings; two openings are clearcuts, while two openings are shelterwood cuts. In the uneven-aged units, research cores consist of four 0.4 ha, two 1.2 ha and two 2 ha canopy openings. The remainder of the research core is given a single tree selection harvest (Kalb & Mycroft, 2013). We were interested in determining if Cerulean Warbler fledglings use the harvested areas in the treatments units once they disperse from their natal territory.

### Nest searching and monitoring

We followed birds closely and noted any individual carrying nest material or food, and then followed the bird to its nest. Observing behavioral cues of Cerulean Warbler pairs is very important in determining the location of the nest (Wagner & Islam, 2014). Detailed notes on nest activities were recorded along with the stage of nesting, such as nest building, incubation, nestlings, or if the nestlings fledged from the nest. Male Cerulean Warblers often “whisper” sing when they are close to their nest and females tend to perform a short free-fall off of their nests, which gave us additional clues as to the approximate location of the nest. Each nest was monitored closely every one to three days for at least a half hour, depending on the stage of the nest. When nestlings were about to fledge (generally day 10 or 11; Buehler, Hamel & Boves, 2013), nests were monitored daily to increase the chances of finding fledglings to band and attach transmitters. A spotting scope was used to observe the nest closely to record detailed notes on nest activities.

### Capture, banding, and auxiliary marker attachment

For target-banding (targeting only Cerulean Warblers), we set up a mist-net on the ground, or used a canopy net suspended from a large horizontal tree limb. At the base of the mist-net, in the middle section of the net, we placed a speaker with an MP3 player and played a Cerulean Warbler song or call to entice the bird into the net. Occasionally, we played an Eastern Screech-Owl (*Megascops asio*) call to capture a bird. Fledglings were often captured by hand near the ground or by using an extending pole with an attached net. Once captured, the Cerulean Warbler was banded with an aluminum United States Geological Survey (USGS) numbered leg band followed by a combination of color bands. An Indiana Department of Natural Resources (IDNR) state collecting permit and a federal bird banding permit (Permit #21781) issued by the USGS were obtained to capture birds and place auxiliary markers (i.e., radio-transmitters, geolocators, and color bands) on Cerulean Warblers. Permission was also granted through the Ball State University Institutional Animal Care and Use Committee (IACUC) to capture and band birds (IACUC approval 437484-4).

When nestlings fledged from the nest, we attempted to capture at least one fledgling or one adult bird from each nest and equipped it with a radio-transmitter (Blackburn Transmitters, Nacogdoches TX, USA). In 2015, radio-transmitters weighed 0.25 g without a harness. These transmitters were only designed to last for five to seven days. For the 2016 and 2017 field seasons, we attached transmitters that lasted up to 22 days, and weighed 0.33 g without the harness. We attached transmitters using the Rappole & Tipton (1991) method with modifications designed by Streby et al. (2015a). Harnesses for transmitters were made of an elastic sewing thread, which would degrade allowing the transmitter to fall off of a bird after a brief period of time (about 40 days; Streby et al., 2015a). The radio-transmitter was glued to the figure eight harness using Loctite super glue.

### Tracking and observations

Radio-tracking started the day following capture. A TRX-1000 receiver and a three-element folding yagi antenna (Wildlife Materials, Inc.) were used to track fledglings once per day. We followed each fledgling Cerulean Warbler for a half hour after initially locating the individual. Locations were recorded on data sheets and in Global Positioning System (GPS) devices. Behavioral observations were also recorded on datasheets. Birds were tracked using the honing method, which uses radio-telemetry to track individuals on foot until the radio-tagged birds are found. Other recorded data included woody plant species where fledglings were perched, approximate height of fledgling from the ground, date, time, and weather conditions such as approximate temperature, precipitation and whether it was sunny or cloudy. Weather data were obtained from the Weather Channel via a smartphone application. Our receiver was not waterproof; therefore, if it rained for an entire day, or if lightning was in the area, tracking was not carried out for that day.

### Microhabitat sampling

In early July of each year, vegetation data were collected at each fledgling location, and at non-use random points corresponding to each of those locations. We collected data at non-use sites to compare microhabitat characteristics to points where fledgling Cerulean Warblers were tracked. ArcGIS 10.3.1 (Esri, Redlands, CA, USA) was used to determine random vegetation points by creating random points within the respective study site (buffer included). Random points were not allowed within 50 m of a fledgling location (Wagner & Islam, 2014; Barnes, Islam & Auer, 2016). All vegetation data were measured within a 15 meter radius of the center point. The center point was located at precise random point coordinates for non-use points, and at precise points where fledglings were tracked. We recorded the date, point identification (bird ID or random point name/number), closest grid point (from the point count grid system; Islam et al., 2013), and aspect. Slope was calculated using a clinometer, 11.3 m from the center point in the uphill and downhill directions. Canopy and ground cover were measured in the four cardinal directions from 2–10 m from the center point in two meter increments, identified by flags. Both measures of cover were presence/absence; canopy cover was determined using a densitometer and ground cover was determined by recording presence/absence of green vegetation (where the flag entered the soil) at every two m intervals (Wagner & Islam, 2014; Barnes, Islam & Auer, 2016). Shrubs were counted and grouped into two categories: <3 cm diameter at breast height (dbh) and 3–10 cm dbh. Shrubs were only measured in a five meter radius of the center point. Mature trees were classified as any woody vegetation >10 cm in dbh and within 11.3 m radius of the center point; all mature trees were measured for dbh. Within the 11.3 m radius, the tallest tree in each quadrant was measured with a Nikon laser 440 rangefinder (Wagner & Islam, 2014). At 15 m from the center point, vertical forest density or stratification was measured using a 2.5 m tall density board that was taped off into five sections. Each section of the density board that was blocked by vegetation was assigned a percent value of cover by the data recorder based on how much of the blocks were covered by live vegetation. Presence of grapevine within the 11.3 m radius was also recorded (Wagner & Islam, 2014).

### Statistical analysis

Analyses were performed in program R (R Core Team, 2015). A Spearman’s correlation test was used to identify auto-correlated variables. A correlation coefficient of 0.60 was used as the ‘cut-off’ point to determine which variables to include in the model (Bakermans, Rodewald & Vitz, 2012; Vitz & Rodewald, 2011). All continuous variables were scaled in the dataset to standardized *z*-scores.

A generalized-linear model with mixed effects was used to account for non-independence among the samples (each bird was tracked multiple times). A model for each combination of variables was created and included the mixed-effect function into each model. Once each model was completed, summary statistics were generated for each model to obtain Akaike Information Criterion (AIC) values. These values were transformed to second-order AIC (AICc) values to account for small sample sizes. A table of the AICc values was produced and all models with values of ≤2.0 were selected as equally plausible models. Model averaging was used to identify which variables in the accepted models were of utmost importance. Model-averaged coefficients were used to make predictions on presence of fledgling sites based on every covariate in the selected models. The above methods were used to compare fledgling Cerulean Warbler micro-habitat characteristics to random non-use locations.

## Results

From 2015–2017, ten fledgling Cerulean Warblers from different nests were tracked via radio-telemetry. Seven radio-transmitters were attached to fledgling Cerulean Warblers. Two radio-transmitters were placed on adult males, and one was placed on an adult female as a proxy for tracking adults to locations of fledglings. Adults were used as a proxy because fledgling Cerulean Warblers can be near impossible to capture in some instances. Also, the dates of tracking fledgling were during a period of time right after fledging when young are dependent on adults for food and not independent to start moving on their own. When adult birds were tracked instead of juveniles, fledgling locations were recorded where the juveniles were found being fed by the adults. The distances moved by each bird, and the number of days tracked varied among individuals. The average distance traveled by fledgling Cerulean Warblers from their nest during tracking was 355.2 m (range 12–1,396 m), and the average number of days individuals were tracked was 10 (range 1–22 days; Table 1). Of the 10 fledglings that were tracked during this study, one may have either lost its radio-transmitter, or died of unknown causes. In this instance, the radio-transmitter was tracked to the same tree for three days, but no radio-transmitter was recovered.

**Table 1:** Distances and the number of days fledgling Cerulean Warblers were tracked in 2015, 2016, & 2017. The line below bird #3 separates the different weighted radio- transmitters used.

Bird	Study unit	Furthest distance traveled from nest (m)	Distance from nest on last observation (m)	# of days tracked
1^*^	8	266	243	6
2^*^	6	NA	97^**^	4
3^*^	8	12	12	1
4	8	1,393	1,214	14
5	5	339	167	17
6	8	396	396	3
7	8	164	164	22
8	8	72	28	16
9	4	104	104	7
10	8	704	704	8
Averages		383.3	312.9	9.8

**Notes.**

* Adult Cerulean Warblers that were used as a proxy to track fledgling Cerulean Warblers.

** Distance between day one and day four of tracking because nest location was unknown.

**Figure 1:**
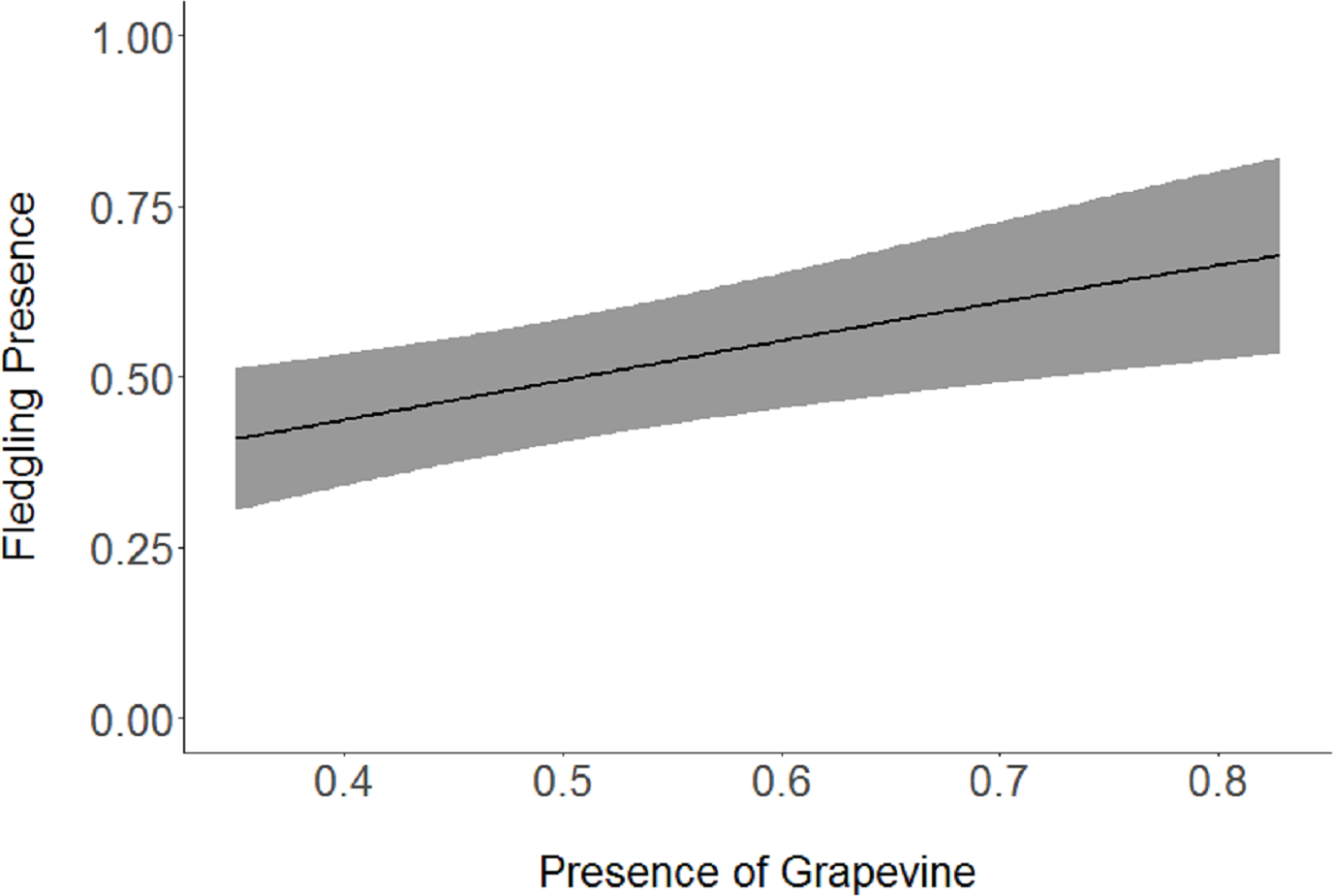
Cerulean Warbler fledglings were positively associated with presence of grapevines at Morgan-Monroe and Yellowwood state forests, Indiana, 2015–2017. Actual values are presented on *x*-axis. The gray areas represent the 95% confidence intervals.

**Figure 2:**
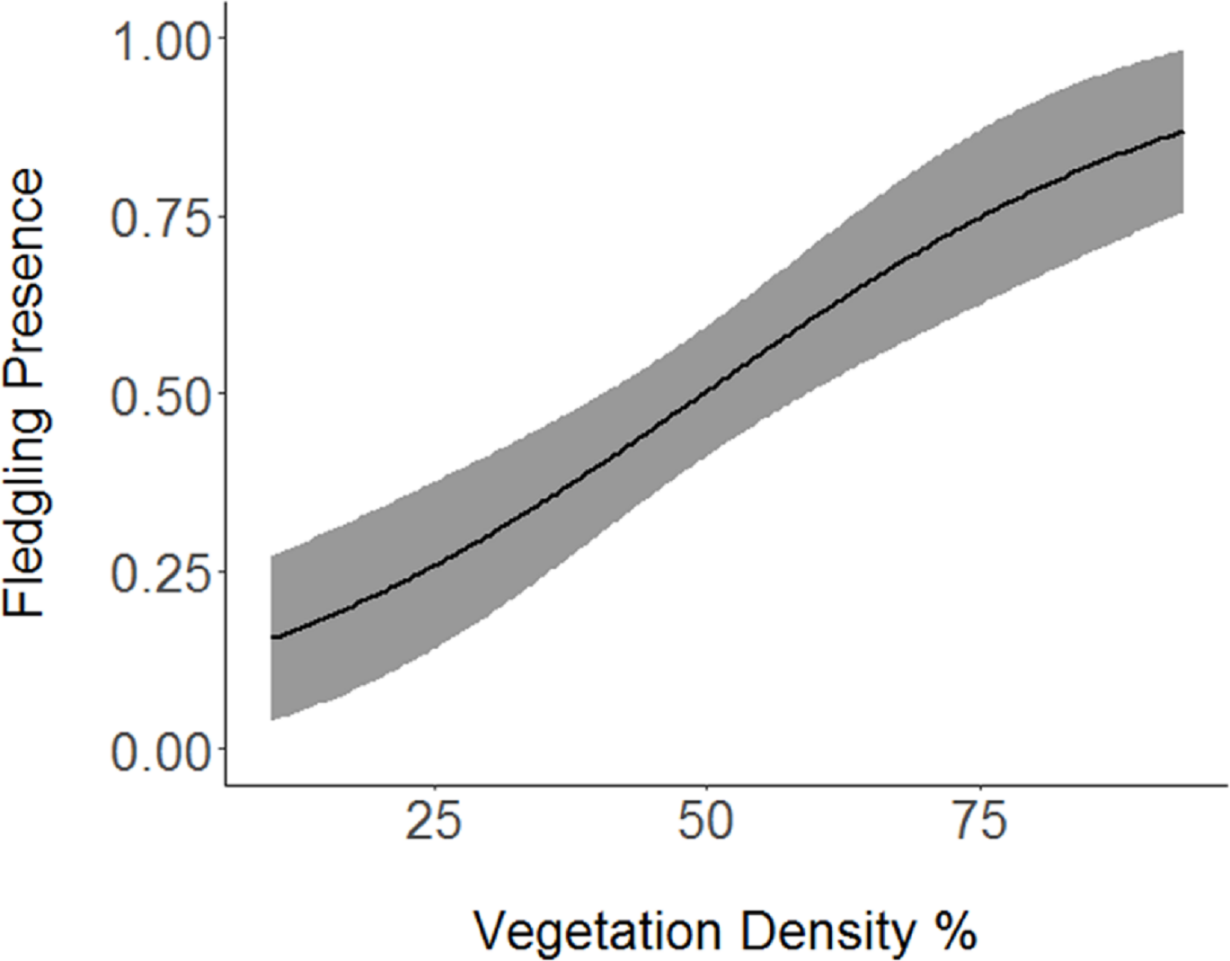
Cerulean Warbler fledglings were positively associated with greater vegetation density at Morgan–Monroe and Yellowwood state forests, Indiana, 2015–2017. Actual values are presented on the *x*-axis. The gray areas represent the 95% confidence intervals.

**Figure 3:**
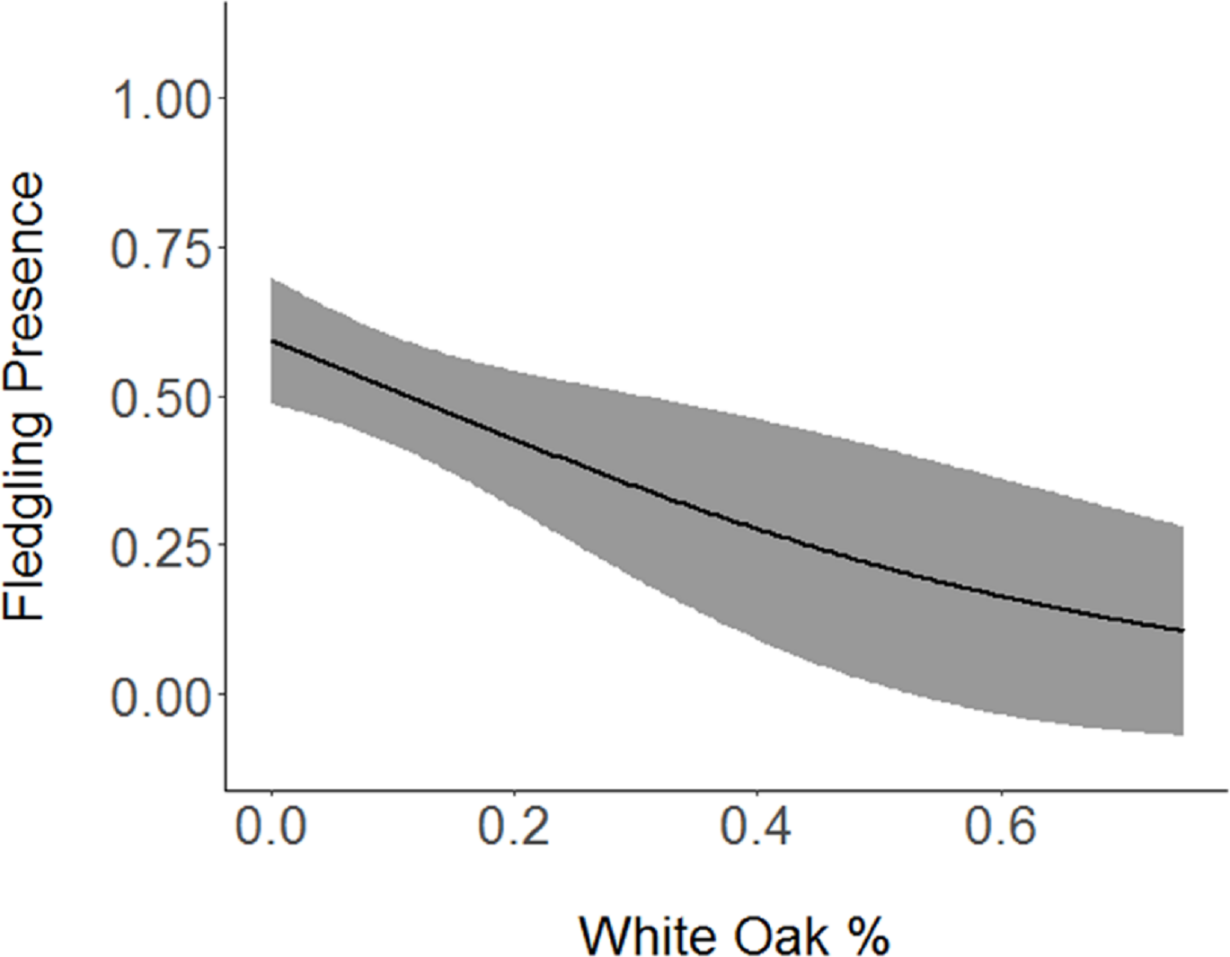
Cerulean Warbler fledglings were negatively associated with white oak abundance at Morgan–Monroe and Yellowwood state forests, southern Indiana, 2015–2017. Actual values are presented on the *x*-axis. The gray areas represent the 95% confidence intervals.

**Figure 4:**
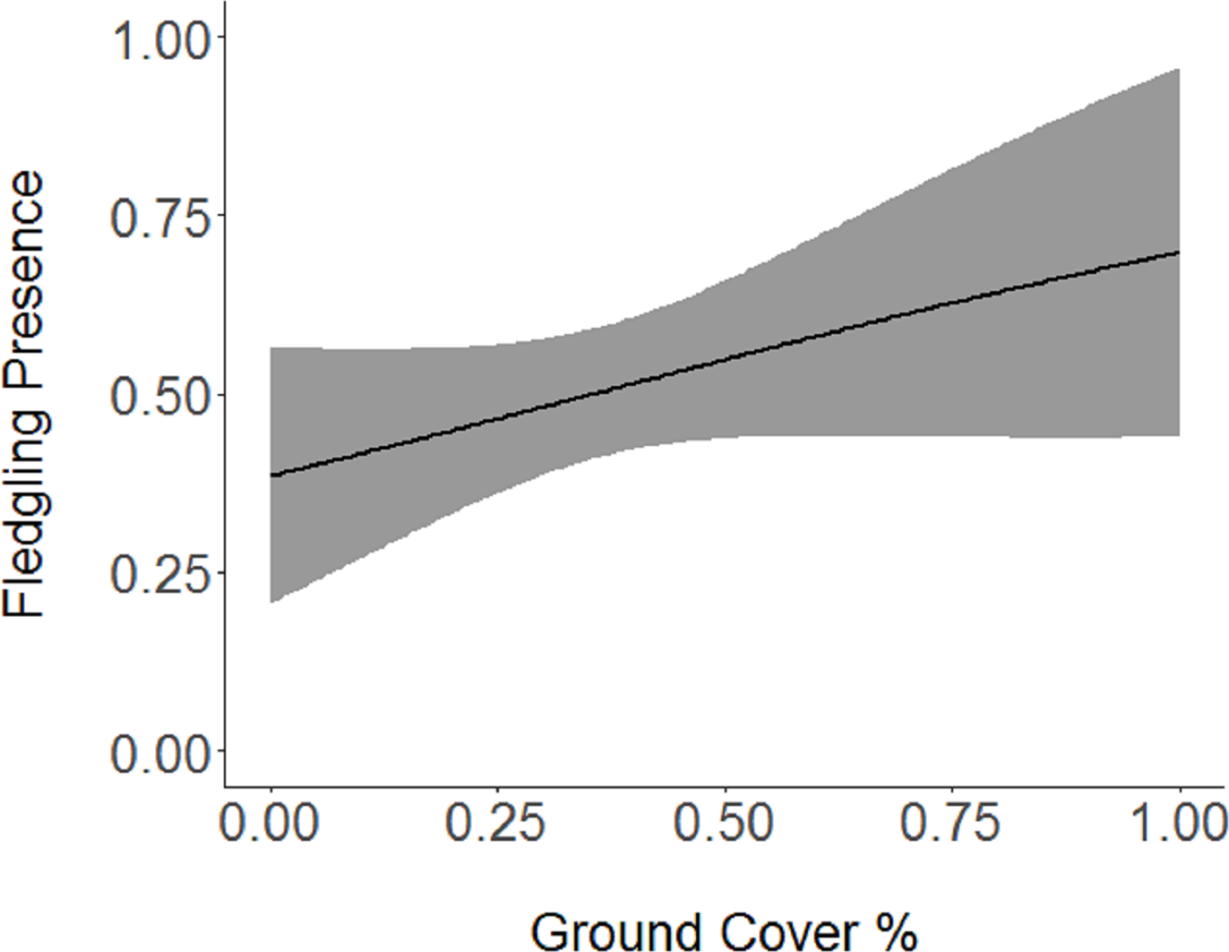
Cerulean Warbler fledglings were positively associated with an increase in ground cover at Morgan–Monroe and Yellowwood state forests, southern Indiana, 2015–2017. Actual values are presented on the *x*-axis. The gray areas represent the 95% confidence intervals.

**Figure 5:**
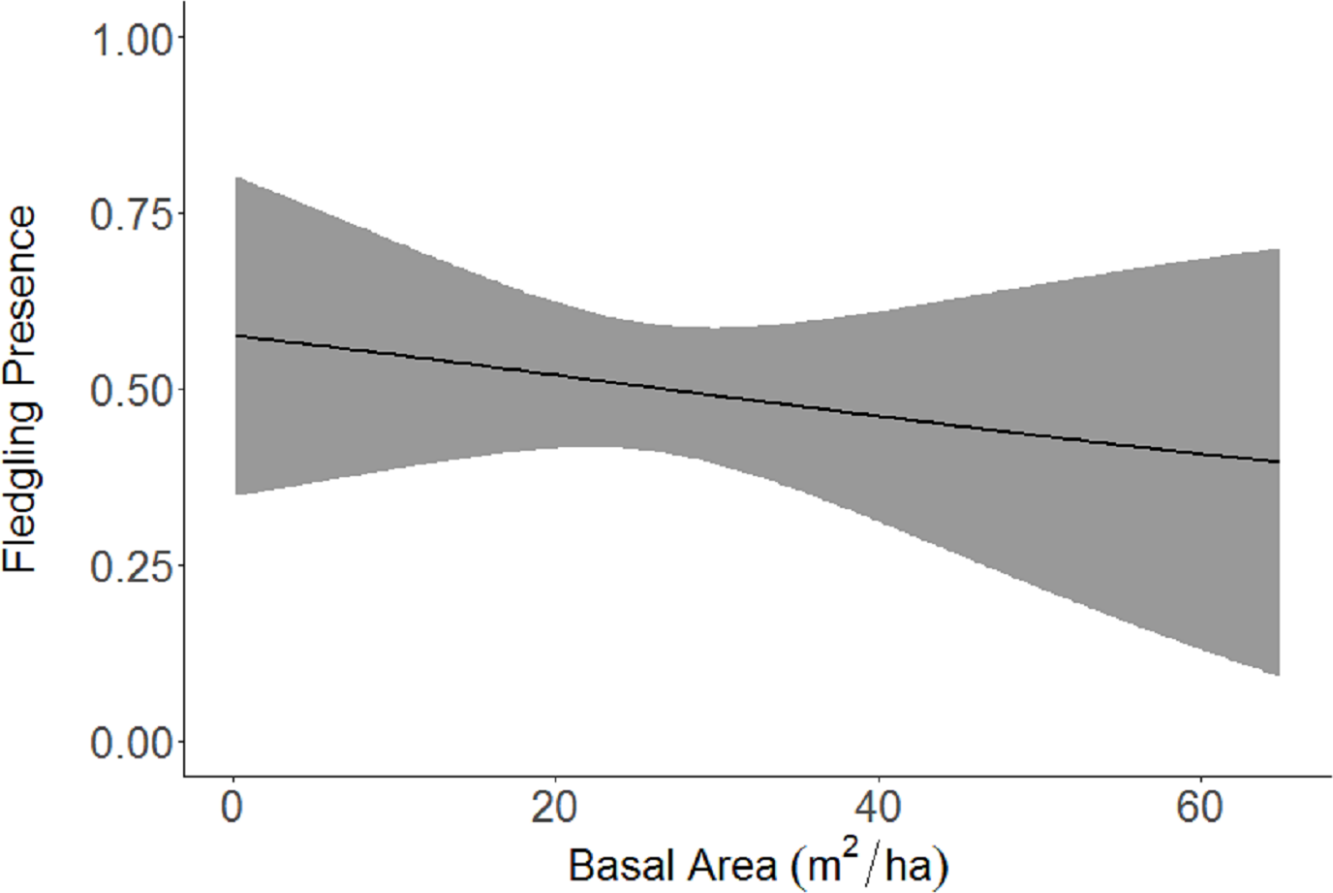
Cerulean Warbler fledglings presence decreased as basal area increased at Morgan–Monroe and Yellowwood state forests, southern Indiana, 2015–2017. Actual values are presented on the *x*-axis. The gray areas represent the 95% confidence intervals.

Based on the results of the Spearman’s correlation analysis, we reduced the number of variables from 14 to 10. Based on ΔAICc values ≤ 2.0, nine models were selected (Table 2). Variables included in the nine models were grapevine presence, percent of white oak, vegetation density, ground cover, basal area, canopy cover, aspect, and the percent of nest tree species nearby. Fledgling presence was strongly correlated with presence of grapevines (1.11; Table 3; Fig. 1) and vegetation density (0.85; Fig. 2), and negatively correlated with presence of white oak abundance (−0.52; Fig. 3). Grapevine, white oak abundance, and vegetation density were found in all ten models. Fledgling presence was positively correlated with ground cover (0.31; Fig. 4) and ground cover was important in 7 models. In contrast, fledgling presence was negatively correlated with basal area (−0.12; Fig. 5). Fledgling presence was positively correlated with canopy cover (0.03), but negatively correlated with aspect (−0.02) and found in only two models. Fledgling presence was negatively correlated with the abundance of mature trees (tree species used by Cerulean Warblers for nesting; −0.01) and it was only found in one model.

**Table 2:** Models, along with AICc values, degrees of freedom, and weights for presence of fledgling Cerulean Warbler locations compared to random vegetation points.

**Models**	**ΔAICc**	**k**	**Weight**
Grapevine + white oak + vegetation density + ground cover	0.00	6	0.056
Grapevine + basal area +white oak + vegetation density + ground cover	0.11	7	0.053
Grapevine + white oak + aspect + ground cover + vegetation density	1.49	7	0.027
Grapevine + white oak + canopy cover + vegetation density + ground cover	1.50	7	0.027
Grapevine + basal area + white oak + vegetation density	1.50	6	0.027
Grapevine + basal area + white oak + canopy cover + vegetation density + ground cover	1.68	8	0.024
Grapevine + basal area + white oak + aspect + vegetation density + ground cover	1.71	8	0.024
Grapevine + nest tree spp. + white oak + vegetation density + ground cover	1.84	7	0.023
Grapevine + white oak + vegetation density	2.00	5	0.021

**Table 3:** Model-averaged coefficients (full average) for fledgling Cerulean Warbler versus random fledgling point models.

	Estimate	Std. Error	Adjusted SE	*Z* value	*Pr*(<|*z*|)	Importance	*N* models
Intercept	−0.374778	0.219248	0.220680	1.698	0.08945	–	–
Grapevine	1.113135	0.400175	0.402766	2.764	0.00571	1.00	9
White oak	−0.521155	0.216475	0.217883	2.392	0.01676	1.00	9
Vegetation density	0.847358	0.206461	0.207786	4.078	0.00005	1.00	9
Ground cover	0.307428	0.222285	0.223177	1.378	0.16836	0.83	7
Basal area	−0.120075	0.181616	0.182185	0.695	0.50984	0.46	4
Aspect	−0.024980	0.091557	0.091955	0.272	0.78589	0.18	2
Canopy cover	0.025701	0.093966	0.094375	0.272	0.78537	0.18	2
Nest tree spp.	−0.008155	0.058678	0.058977	0.138	0.89002	0.08	1

## Discussion

This study is the first to examine where Cerulean Warbler fledglings disperse once they leave the nest. Based on our research findings, vegetation density is a key factor that determines where adult Cerulean Warblers take their young after leaving the nest. Whether it is on the ground, in the shrub layer, or in the canopy, these birds are favoring areas of greater vegetation density. Of 97 fledgling locations surveyed, 51.5% of the vegetation plots had grapevine. However, only 18.6% of random vegetation surveys had grapevine. Grapevine provides excellent cover, and it is an important habitat component in fledgling habitats, likely because it offers protection from predators. Fledglings, especially when less than one week out of the nest, do not typically move very far, and are not strong fliers. Therefore, staying hidden in thick vegetation is beneficial to their survival. Areas that have high vegetation densities in our study sites include clearcuts, patch-cuts, and riparian areas. Adult Cerulean Warblers have been caught in clearcuts in our study sites during the fledgling period (P Ruhl, pers. comm., 2015), and we have observed adult and fledgling Cerulean Warblers in patch-cuts at our sites. As suggested by other studies, fledgling songbirds may utilize these areas after leaving the nest to avoid predation (Vitz & Rodewald, 2006; Vitz, Rodewald & VegaRivera, 2007; Stoleson, 2013).

Based on previous research, nesting habitats differ from post-fledging habitats in some Passerine species (Akresh et al., 2009; Porneluzi et al., 2014). For example, forest-interior Passerines, such as the Black-throated Blue Warbler (*Setophaga caerulescens*) and Ovenbird (*Seiurus aurocapilla*) used clearcuts within a forest matrix during the fledging period (Porneluzi et al., 2014; Stoleson, 2013; Streby et al., 2011). In other species, such as the Golden-winged Warbler, fledglings moved from early successional habitats to forested habitats during the post-fledging period (Streby et al., 2015b; Streby, Peterson & Andersen, 2016). In Passerines, parents will often lead fledglings to riparian forests after their nestlings fledge (Akresh et al., 2009). Proposed explanations for these habitat changes from the nesting and fledgling periods include, higher biomass of insects or fruit, and greater protection from predators (Vitz, Rodewald & VegaRivera, 2007; Akresh et al., 2009; Stoleson, 2013). Similar to our research on Cerulean Warblers, Anders, Faaborg & Thompson III (1998) found that fledgling Wood Thrushes (*Hylocichla mustelina*) dispersed to sites where forests consisted of a dense understory with thick ground cover. Vitz & Rodewald (2006) also found that smaller clearcuts resulted in a greater abundance of forest-interior songbirds than larger clearcuts.

At our sites, Cerulean Warblers often forage in white oak during the nest building, egg-laying, and nestling stages (CD Delancey, pers. obs., 2015–2017; MacNeil, 2010; Auer et al., 2016). Fewer mature trees of preferred nest tree species were present in areas with fledglings. White oak, the main nest tree species used by Cerulean Warblers, was more common in random sites than in areas where fledglings were found. Cerulean Warblers may place territories around areas with clumped white oak distributions. However, once the nestlings fledge, the birds move beyond the territory boundaries (12–1,396 m [from their nest]) where white oak was found to be less common.

Canopy cover was slightly higher at fledgling sites, which is beneficial for young birds that are more vulnerable to predation (Vitz & Rodewald, 2006; Vitz, Rodewald & VegaRivera, 2007; Stoleson, 2013). During the fledgling period, it would be advantageous to move into areas with a higher abundance of insects and areas of dense vegetative growth, which are both characteristic of riparian areas (Akresh et al., 2009). In some instances, fledglings were found in riparian areas. For example, our control units do not have patch-cuts or clearcuts but are characterized by riparian areas. These areas often possess many shrubs and a dense canopy across all strata. Akresh et al. (2009) suggested that songbirds, especially juveniles, preferred riparian areas during the post-fledging period due to higher vegetation densities and an abundance of food. In Ontario, insect abundance was found to be higher in riparian areas compared to upland sites from 16 June-28 July, which correlates to the post-fledging period for many songbirds (Mosley, Holmes & Nol, 2006).

There was a slight negative relationship with presence of fledglings and southwest aspect. Aspect was only found in two models when fledgling locations were compared to random locations and therefore, may not be as important as other variables that showed stronger associations with fledgling presence. There was less basal area in areas where fledglings were present, which would allow for a denser shrub layer. A study in West Virginia examined microhabitat characteristics of vegetation, soil, and climate with respect to aspect and found that afternoon temperatures on west and southwest-facing slopes were about 4.86 °C warmer than on north and east-facing slopes (Desta et al., 2004). Before nestlings fledge, they need to thermoregulate themselves; therefore, moving to warmer locations will help them regulate their body temperatures without expending additional energy. Also, the relative humidity on these western and southwestern slopes was found to be about 25% less than that on north and eastern slopes. Basal area also averaged 7.84 m^2^/ha higher on north and eastern slopes (Desta et al., 2004).

In three instances, fledglings moved southward and downstream from their nest locations (Fig. S1). Riparian areas are often corridors for migration with areas of dense cover to protect fledglings from predators; riparian areas also offer ample food to help fledglings grow fast. The Cerulean Warbler is a single-brooded species that leaves its breeding sites in southern Indiana by the beginning of August (C.D. Delancey, pers. obs.); it is possible that once the young fledge, family groups move southward, following streams. In some instances, fledglings moved up slope, and in a northward direction (Figs. S1, S2). One of these individuals chose to spend some time in a pine (*Pinus spp*.) stand, which provided abundant dense cover (Fig. S1). Some birds chose to stay relatively close to their nests (Figs. S1, S3 and S4). In these instances, surrounding areas near the nest tree had higher vegetation densities than riparian areas, including harvested areas nearby or areas where grapevines had spread into the canopy. In the case of one fledgling that was tracked in a control unit, the area surrounding the nest site received a light harvest a few years ago, resulting in growth of shrubs and saplings in the understory (Fig. S3). Harvest was allowed in this area because it was in the buffer zone of the HEE study sites.

Many observations of nestlings at the time of fledging demonstrated that nestlings could leave the nest and still stay high up in the canopy, making it impossible to capture. We noticed that nestlings that flapped their wings while on the nest were less likely to be caught and stayed high up in the canopy. Only fledglings that came within ∼7 m of the forest floor could be captured for this study. These nestlings may have been the weaker individuals in the nest, or maybe nestlings that left the nest prematurely. Once on the ground, fledglings worked their way back into the canopy within a couple of days.

We were unable to compare fledgling movements among the different study sites due to our sample size of 10 fledglings. Additionally, the data on fledglings is skewed toward uneven-aged study sites because one of the sites (unit 8) has the greatest relative abundance of Cerulean Warblers compared to the other sites. Seven of the ten fledgling Cerulean Warblers tracked were located within unit 8. One fledgling was tracked in unit 6 (an uneven-aged unit), and two fledglings were tracked in control units (units 4 and 5). Both Cerulean Warbler fledglings tracked in control units were in disturbed areas along forest roads or in an area within the buffer that was recently harvested. The areas where these two fledgling Cerulean Warblers were tracked resembled an uneven-aged forest stand.

### Management recommendations

Fledgling locations were found to have greater vertical vegetation density, which is indicative of an uneven-aged forest structure. Although, Cerulean Warblers rely on grapevine, which is usually considered to grow best in even-aged forest stands and known to reduce timber quality, growth of grapevines should be encouraged to benefit Cerulean Warblers. Grapevine is vital for nesting Cerulean Warblers, as it is for fledglings as cover. Riparian corridors should be protected to allow for greater development of canopy cover for Cerulean Warbler fledglings. A light thinning within a forest stand, that conserves white oak, will allow the understory to develop, while at the same time it is important to maintain a high canopy density which are both important habitat components for fledgling Cerulean Warblers. By decreasing the basal area of mature trees at these sites, a mixed-age forest stand can be produced. A forest with an established canopy, along with many mid-story trees and shrub cover on the ground, will benefit Cerulean Warbler fledglings that are hiding from predators; maintaining structural diversity is key to managing for declining populations of Cerulean Warblers. More research will need to be completed to determine how large of an area to manage for Cerulean Warblers and their fledgling habitat. Our limited data shows that the distance traveled after leaving the nest can vary (12–1,396 m in 1–22 days post-fledging). These data, however, may only be applicable to the Cerulean Warbler population in Indiana and nearby. More research across the Cerulean Warbler breeding range can help address if there are any region-specific variations among fledgling habitats.

## Conclusion

This is the first study that examined habitat use by fledgling Cerulean Warblers. We found that fledgling habitat differed from other habitats that Cerulean Warblers utilize during the breeding season. Clearcuts or smaller patch-cuts near breeding sites can also benefit Cerulean Warblers in the post-fledging period as areas with plentiful food and protection from predators. Identifying the different vegetation types that Cerulean Warblers use throughout the breeding season can best inform natural resource personnel on how to manage forests to meet the habitat requirements of this declining songbird. However, continued research across the breeding distribution of Cerulean Warblers can best determine if our results are regional preferences, or if our results are similar throughout their range.

##  Supplemental Information

10.7717/peerj.7358/supp-1 Figure S1 Map of seven different fledgling Cerulean Warblers (1, 3, 4, 6, 7, 8, 10) from different nests This study site was located in one of our “uneven-aged forests” in Yellowwood State Forest, Brown County, Indiana (May-July 2015-2017). Sources: Esri, DigitalGlobe, Earthstar Geographics, CNES/Airbus DS, GeoEye, USDA FSA, USGS, Aerogrid, IGN, IGP, and the GIS User Community. (C) ESRI.

10.7717/peerj.7358/supp-2 Figure S2 Map of fledgling #2 in an “even-aged forest” in Yellowwood State Forest, Brown County, Indiana (June 2015) We did not know the nest location where this fledgling came from, but it appeared to be a local fledgling. D1 shows the starting location, and D4 shows where we lost contact with the fledgling. Sources: Esri, DigitalGlobe, Earthstar Geographics, CNES/Airbus DS, GeoEye, USDA FSA, USGS, Aerogrid, IGN, IGP, and the GIS User Community. (C) ESRI.

10.7717/peerj.7358/supp-3 Figure S3 Map of fledgling #9 in a control site in Morgan-Monroe State Forest, Monroe County, Indiana (June-July 2017) Sources: Esri, DigitalGlobe, Earthstar Geographics, CNES/Airbus DS, GeoEye, USDA FSA, USGS, Aerogrid, IGN, IGP, and the GIS User Community. (C) ESRI.

10.7717/peerj.7358/supp-4 Figure S4 Map of fledgling #5 in a control site in Yellowwood State Forest, Brown County, Indiana (June 2016) Sources: Esri, DigitalGlobe, Earthstar Geographics, CNES/Airbus DS, GeoEye, USDA FSA, USGS, Aerogrid, IGN, IGP, and the GIS User Community. (C) ESRI.

## Additional Information and Declarations

### Field Study Permissions

The following information was supplied relating to field study approvals (i.e., approving body and any reference numbers): An Indiana Department of Natural Resources (IDNR) state collecting permit and a federal bird banding permit (Permit #21781) issued by the USGS were obtained to capture birds and place auxiliary markers (i.e., radio-transmitters, geolocators, and color bands) on Cerulean Warblers.

### Data Availability

The following information was supplied regarding data availability: Data are available at Figshare: Delancey, Clayton (2019): Habitat Use by Fledgling Cerulean Warblers. figshare. Dataset. https://doi.org/10.6084/m9.figshare.8091197.v1.

## References

[ref-1] Akresh AE, Dinse K, Foufopoulos J, Schubel SC, Kowalczyk T (2009). Passerine breeding and post-fledgling habitat use in riparian and upland temperate forests of the American Midwest. The Condor.

[ref-2] Anders AD, Faaborg J, Thompson III FR (1998). Postfledging dispersal, habitat use, and home-range size of juvenile Wood Thrushes. The Auk.

[ref-3] Auer SA, Islam K, Wagner JR, Summerville KS, Barnes KW (2016). The diet of Cerulean Warbler (*Setophaga cerulea*) nestlings and adult nest provisioning behaviors in Southern Indiana. Wilson Journal of Ornithology.

[ref-4] Bakermans MH, Rodewald AD, Vitz AC (2012). Influence of forest structure on density and nest success of mature forest birds in managed landscapes. Journal of Wildlife Management.

[ref-5] Barnes KW, Islam K, Auer SA (2016). Integrating LIDAR-derived canopy structure into cerulean warbler habitat models. Journal of Wildlife Management.

[ref-6] BirdLife International (2019). IUCN red list for birds. http://www.birdlife.org.

[ref-7] Buehler DA, Giocomo JJ, Jones J, Hamel PB, Rogers CM, Beachy TA, Varble DW, Nicholson CP, Roth KL, Barg J, Robertson RJ, Robb JR, Islam K (2008). Cerulean warbler reproduction, survival, and models of population decline. Journal of Wildlife Management.

[ref-8] Buehler DA, Hamel PB, Boves T, Poole A (2013). Cerulean Warbler (*Setophaga cerulea*), the birds of North America online.

[ref-9] Burke AD, Thompson III FR, Faaborg J (2017). Variation in early-successional habitat use among independent juvenile forest breeding birds. Wilson Journal of Ornithology.

[ref-10] Campbell SP, Witham JW, Hunter Jr ML (2007). Long-term effects of group-selection timber harvesting on abundance of forest birds. Conservation Biology.

[ref-11] COSEWIC (2010). COSEWIC assessment and status report on the Cerulean Warbler *Dendroica cerulea* in Canada.

[ref-12] Desta F, Colbert JJ, Rentch JS, Gottschalk KW (2004). Aspect induced differences in vegetation, soil, and microclimate characteristics of an Appalachian watershed. Castanea.

[ref-13] Hamel PB (2000a). Cerulean warbler status assessment.

[ref-14] Hamel PB, Poole A, Gill F (2000b). Cerulean Warbler (*Dendroica cerulea*). Birds of North America, no. 511.

[ref-15] Hamel PB, Dawson DK, Keyser PD (2004). How we can learn more about the Cerulean Warbler (*Dendroica cerulea*)?. Auk.

[ref-16] Holmes RT (2007). Understanding population change in migratory songbirds: long-term and experimental studies of Neotropical migrants in breeding and wintering areas. Ibis.

[ref-17] Indiana General Assembly (2007). Title 312 Natural Resources Commission. Indiana Register. http://www.in.gov/legislative/iac/20070117-IR-312060272EIA.xml.html.

[ref-18] Islam K, Kaminski KJ, MacNeil MM, Young LP, Swihart RK, Saunders MR, Kalb RA, Haulton SG, Michler CH (2013). The Cerulean Warbler in Morgan–Monroe and Yellowwood state forests, Indiana: pre-treatment data on abundance and spatial characteristics of territories. The Hardwood ecosystem experiment: a framework for studying responses to forest management.

[ref-19] Kalb RA, Mycroft CJ, Swihart RK, Saunders MR, Kalb RA, Haulton SG, Michler CH (2013). Indiana forest management history and practices. The Hardwood ecosystem experiment: a framework for studying responses to forest management.

[ref-20] MacNeil MM (2010). Does timber harvesting affect Cerulean Warbler foraging ecology. Master’s thesis.

[ref-21] Martin TE, Finch DM (1995). Ecology and management of Neotropical migratory birds: a synthesis and review of critical issues.

[ref-22] Mosley E, Holmes SB, Nol E (2006). Songbird diversity and movement in upland and riparian habitats in the boreal mixed-wood forest of northeastern Ontario. Canadian Journal of Forest Research.

[ref-23] Porneluzi PA, Brito-Aguilar R, Clawson RL, Faaborg J (2014). Long-term dynamics of bird use of clearcuts in post-fledging period. Wilson Journal of Ornithology.

[ref-24] R Core Team (2015). http://www.R-project.org/.

[ref-25] Rappole JH, Tipton AR (1991). New harness design for attachment of radio transmitters to small passerines. Journal of Field Ornithology.

[ref-26] Robinson SK, Thompson III FR, Donovan TM, Whitehead DR, Faaborg J (1995). Regional forest fragmentation and the nesting success of migratory birds. Science.

[ref-27] Sauer JR, Hines JE, Fallon JE, Pardieck KL, Ziolkowski Jr DJ, Link WA (2012). The North American breeding bird survey: results and analysis 1966–2012.

[ref-28] Stoleson SH (2013). Condition varies with habitat choice in postbreeding forest birds. Auk.

[ref-29] Streby HM, Peterson SM, McAllister TL, Andersen DE (2011). Use of early-successional managed northern forest by mature-forest species during the post-fledging period. The Condor.

[ref-30] Streby HM, McAllister TL, Peterson SM, Kramer GR, Lehman JA, Anderson DE (2015a). Minimizing marker mass and handling time when attaching radio-transmitters and geolocators to small songbirds. The Condor.

[ref-31] Streby HM, Peterson SM, Kramer GR, Andersen DE (2015b). Post-independence fledgling ecology in a migratory songbird: implications for breeding-grounds conservation. Animal Conservation.

[ref-32] Streby HM, Peterson SM, Andersen DE, Streby HM, Andersen DE, Buehler DA (2016). Golden-winged warbler fledgling habitat use and survival in the western Great Lakes region. Golden-winged warbler ecology, conservation, and habitat management.

[ref-33] United States Fish and Wildlife Service (USFWS) (2006). Cerulean Warbler (*Dendroica cerulea*) Fact Sheet. http://www.fws.gov/midwest/es/soc/birds/cerw/pdf/cerw-fctsheet.pdf.

[ref-34] Vitz AC, Rodewald AD (2006). Can regenerating clearcuts benefit mature-forest songbirds? An examination of post-breeding ecology. Biological Conservation.

[ref-35] Vitz AC, Rodewald AD (2011). Influence of condition and habitat use on survival of post-fledging songbirds. The Condor.

[ref-36] Vitz AC, Rodewald AD, Vega Rivera JH (2007). Vegetative and fruit resources as determinants of habitat use by mature-forest birds during the postbreeding period. Auk.

[ref-37] Wagner JR, Islam K (2014). Nest-site selection and breeding ecology of the Cerulean Warbler in Southern Indiana. Northeastern Naturalist.

[ref-38] Weakland CA, Wood PB (2005). Cerulean Warbler (*Dendroica cerulea*) microhabitat and landscape-level habitat characteristics in southern West Virginia. Auk.

