# Supplementary figures and images for: Post-fledging habitat use in a declining songbird
Source: PeerJ. 2019 Aug 30;7:e7358. doi: 10.7717/peerj.7358 (PMC6718152; doi:10.7717/peerj.7358)

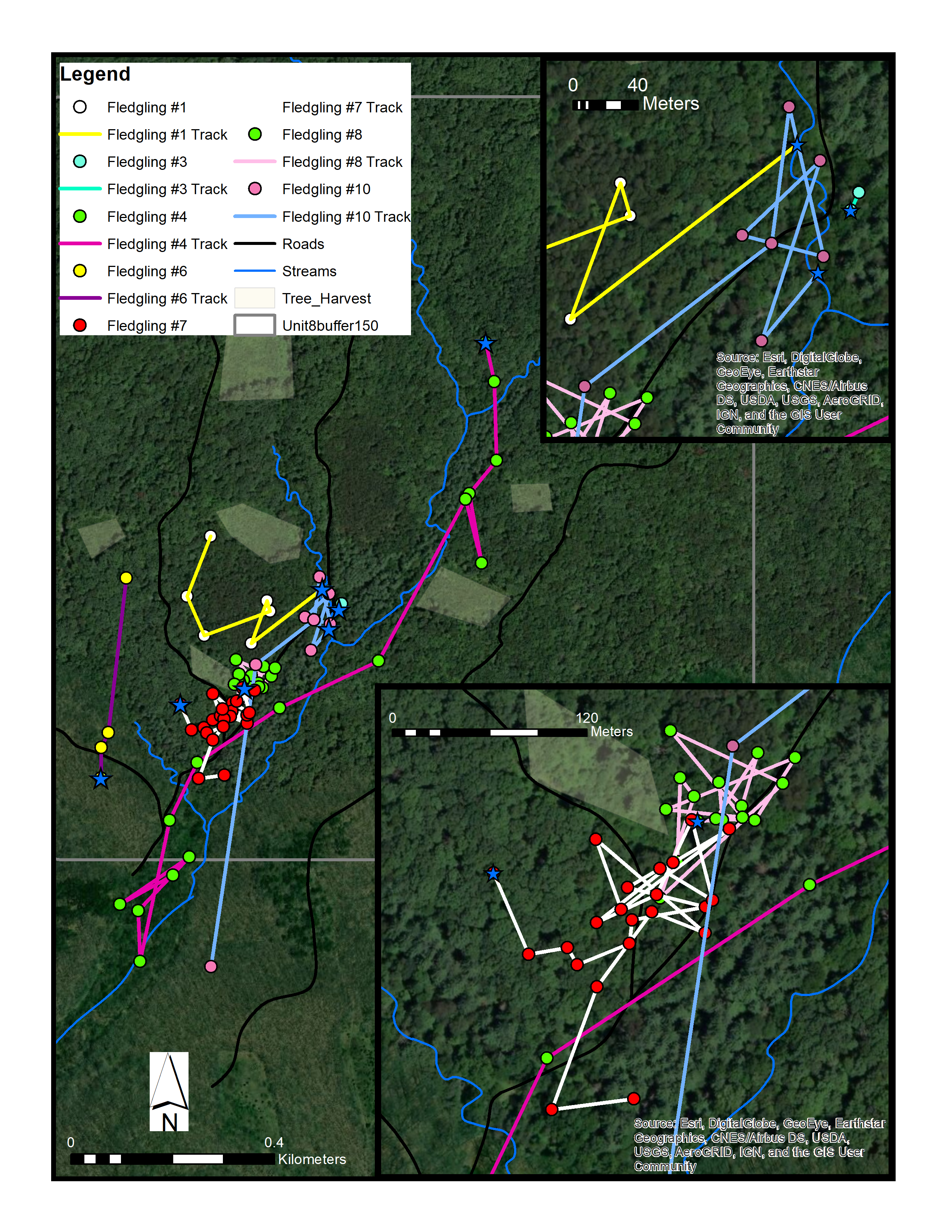

Supplement: Figure S1 — This study site was located in one of our “uneven-aged forests” in Yellowwood State Forest, Brown County, Indiana (May-July 2015-2017). Sources: Esri, DigitalGlobe, Earthstar Geographics, CNES/Airbus DS, GeoEye, USDA FSA, USGS, Aerogrid, IGN, IGP, and the GIS User Community. (C) ESRI. [file peerj-07-7358-s001.png]

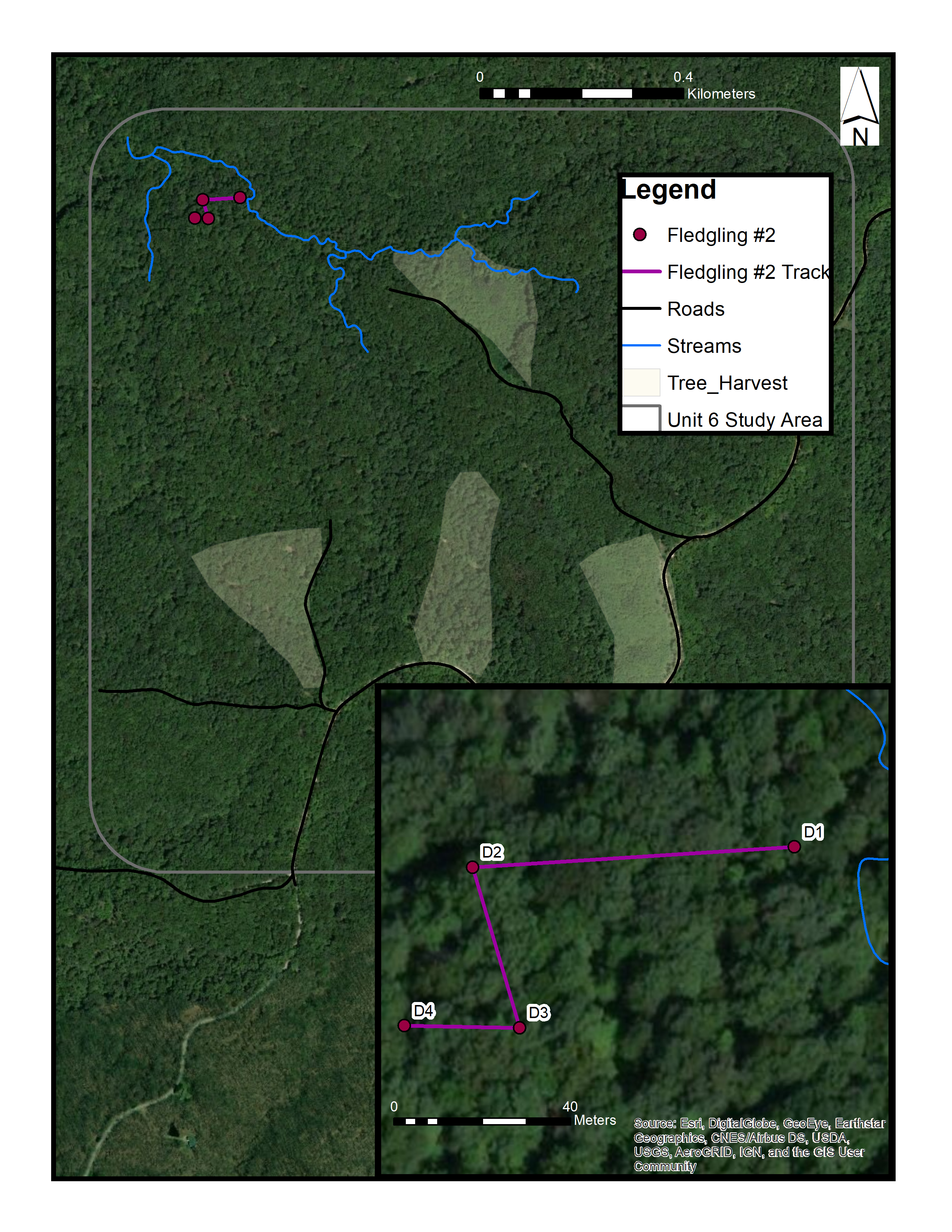

Supplement: Figure S2 — We did not know the nest location where this fledgling came from, but it appeared to be a local fledgling. D1 shows the starting location, and D4 shows where we lost contact with the fledgling. Sources: Esri, DigitalGlobe, Earthstar Geographics, CNES/Airbus DS, GeoEye, USDA FSA, USGS, Aerogrid, IGN, IGP, and the GIS User Community. (C) ESRI. [file peerj-07-7358-s002.png]

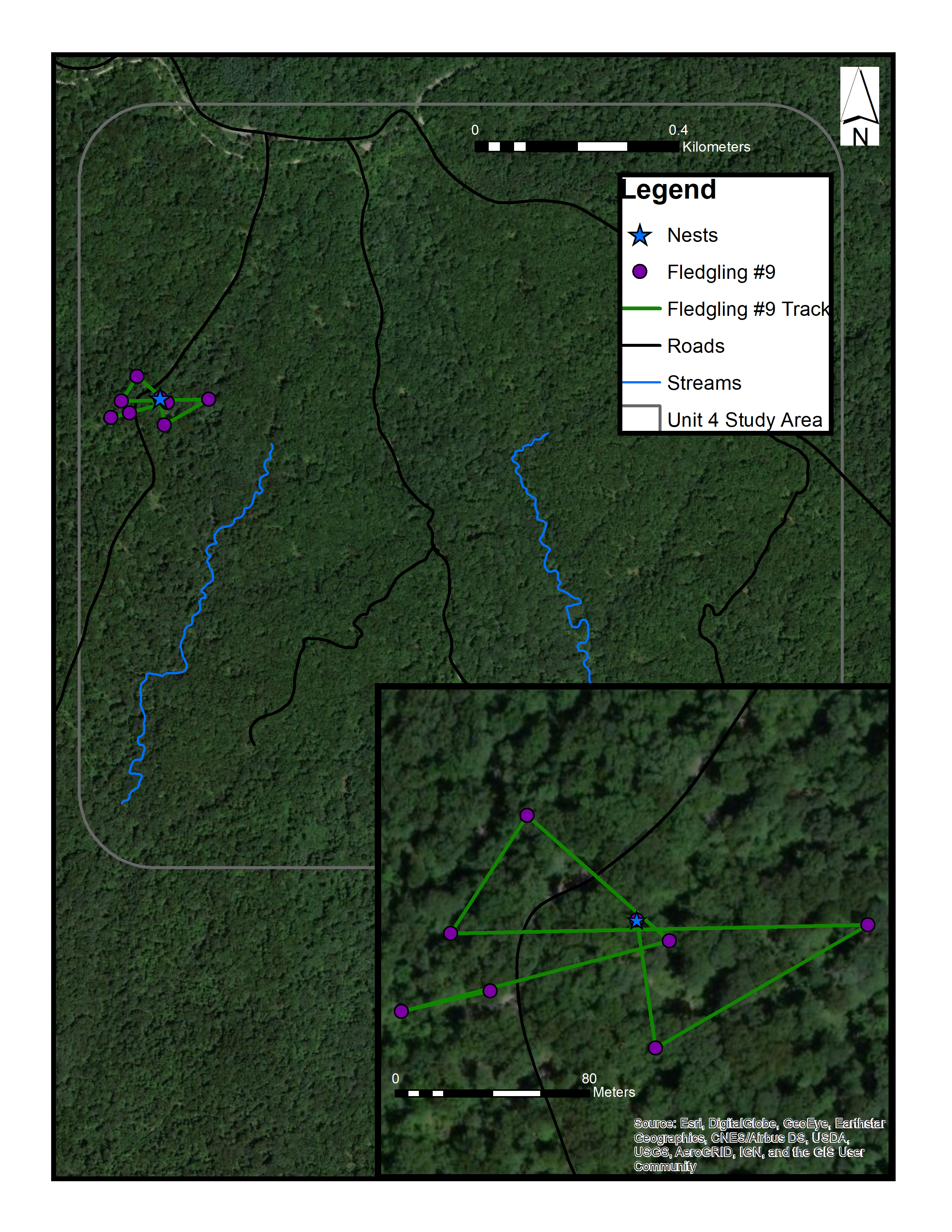

Supplement: Figure S3 — Sources: Esri, DigitalGlobe, Earthstar Geographics, CNES/Airbus DS, GeoEye, USDA FSA, USGS, Aerogrid, IGN, IGP, and the GIS User Community. (C) ESRI. [file peerj-07-7358-s003.png]

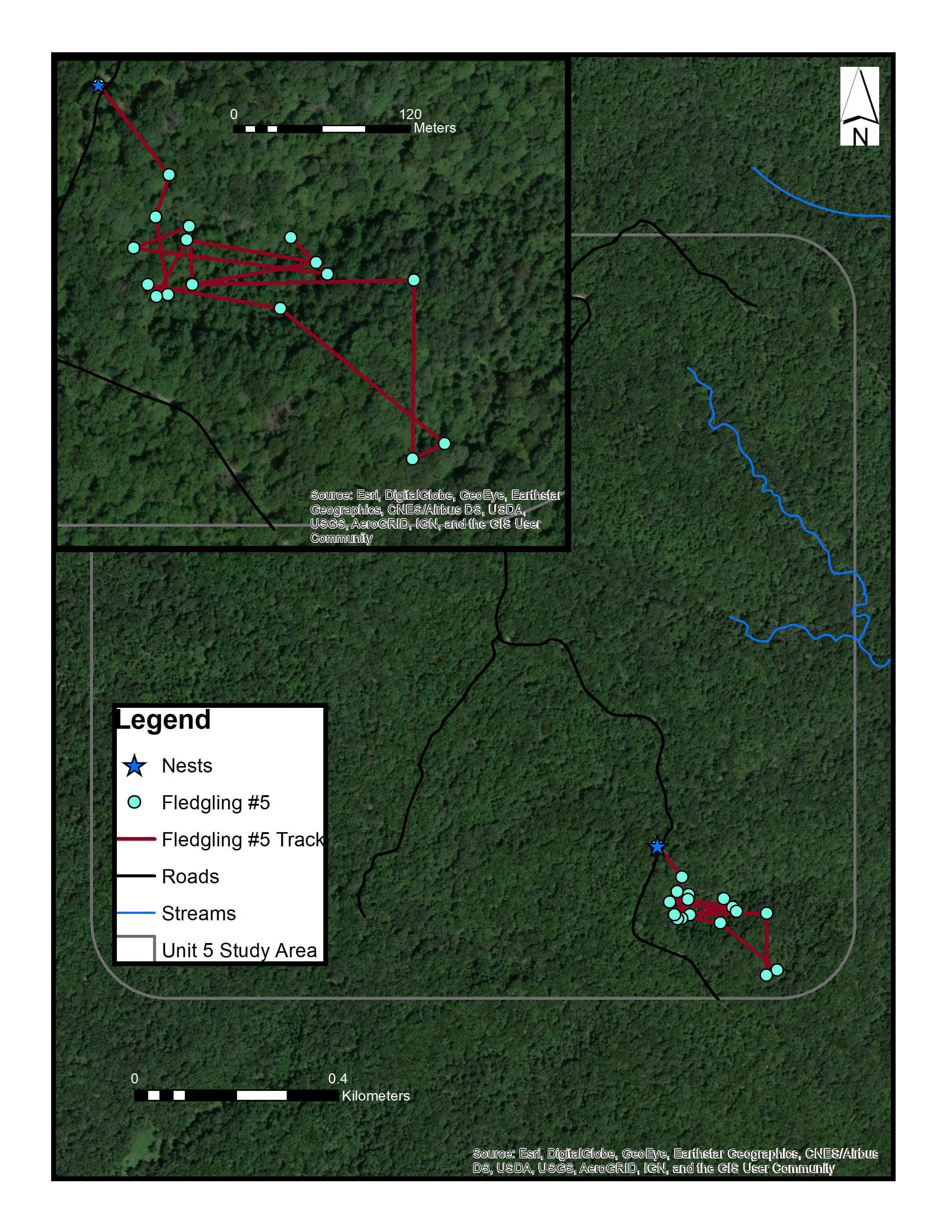

Supplement: Figure S4 — Sources: Esri, DigitalGlobe, Earthstar Geographics, CNES/Airbus DS, GeoEye, USDA FSA, USGS, Aerogrid, IGN, IGP, and the GIS User Community. (C) ESRI. [file peerj-07-7358-s004.png]
